# Decoding the Sphingolipid Landscape of Clear Cell Renal Cell Carcinoma: A Single‐Cell‐Guided Prognostic Model Built With 101 Machine Learning

**DOI:** 10.1155/humu/3249360

**Published:** 2026-05-17

**Authors:** Jinbang Huang, Shunsheng Wang, Yaojun Zhou, Hui Zhang, Yanyuan Zhang, Shi Huang, Haiwei Su, Hongling Zhu, Yuhan Lin, Gang Deng

**Affiliations:** ^1^ Department of General Surgery, The Seventh Affiliated Hospital of Sun Yat-sen University, Shenzhen, China, sysu.edu.cn; ^2^ School of Clinical Medicine, Southwest Medical University, Luzhou, China, swmu.edu.cn

**Keywords:** clear cell renal cell carcinoma, immunotherapy, machine learning, nonnegative matrix factorization (NMF), prognosis prediction, prognostic model, single-cell analysis, sphingolipid metabolism, sphingolipid metabolism–related genes, tumor microenvironment

## Abstract

**Background:**

Clear cell renal cell carcinoma (ccRCC) is the most common histological subtype of kidney cancer and shows marked heterogeneity in progression, metastasis, and therapeutic response. Sphingolipid metabolism has emerged as an important regulator of tumor progression and the tumor microenvironment, but its cell‐state‐specific role in ccRCC remains unclear.

**Methods:**

Public datasets from multiple cohorts were integrated, including single‐cell RNA sequencing data from GEO and bulk transcriptomic and clinical data from Xena, ArrayExpress, and ICGC. Nonnegative matrix factorization was used to resolve cellular heterogeneity and identify sphingolipid metabolism–related characteristic genes across distinct cell subsets. A prognostic model was constructed by screening 101 machine learning combinations and validated in independent cohorts. Additional analyses included immune characterization, pathway enrichment, drug sensitivity prediction, protein–protein interaction network analysis, and mutation profiling.

**Results:**

Single‐cell analysis identified 23 cell clusters representing nine major cell types in ccRCC and further resolved multiple sphingolipid metabolism–related metagene‐defined subclusters within immune and stromal compartments. Based on these features, 101 machine learning combinations were evaluated, and a final 12‐gene prognostic signature was established using the Lasso+SuperPC model. The model showed stable prognostic performance across independent cohorts and outperformed conventional clinical variables. It was also associated with distinct immune features, predicted therapeutic vulnerabilities, and mutation‐related genomic characteristics.

**Conclusion:**

This study provides a single‐cell‐guided framework for understanding sphingolipid metabolism–related heterogeneity in the ccRCC microenvironment and establishes a 12‐gene prognostic signature with potential value for risk stratification and therapeutic prioritization.

## 1. Introduction

Clear cell renal cell carcinoma (ccRCC), constituting approximately 80% of renal malignancies, stands as one of the most prevalent kidney cancers [1, 2]. As the second leading malignancy within the urinary system, its incidence has been escalating annually, presenting a formidable threat to patient survival due to its high recurrence and mortality rates and imposing substantial economic burdens on healthcare systems [3–5]. Despite advances in surgery, targeted therapy, and immunotherapy, clinical outcomes among patients with ccRCC remain highly heterogeneous, particularly with respect to disease progression, metastatic potential, and therapeutic responsiveness [6, 7]. Current prognostic assessment for ccRCC still relies largely on clinicopathological features, which may be insufficient to capture the molecular and microenvironmental diversity that underlies variable patient outcomes [8, 9]. Therefore, there is a pressing need to identify biologically informative markers that can improve risk stratification and support more individualized management of ccRCC.

Sphingolipids, encompassing sphingomyelins, cerebrosides, and gangliosides, represent a crucial class of biological lipids integral to cell membrane composition, where they play a pivotal role in preserving structural integrity and functional stability [10–13]. Beyond their structural contributions, sphingolipids are vital regulators of cellular processes, including signaling, proliferation, and apoptosis, all of which are essential for maintaining physiological function and metabolic equilibrium [14]. Emerging evidence has highlighted the significant role of sphingolipid metabolic dysregulation in the pathogenesis and progression of various cancers [15, 16]. Disruptions in sphingolipid metabolism can lead to aberrant accumulation or depletion of these lipids, compromising cell membrane architecture, impairing cellular exchange and signaling pathways, and thereby facilitating tumorigenesis and progression [17, 18]. Moreover, sphingolipid metabolism exerts considerable influence within the tumor microenvironment (TME), modulating interactions between tumor cells and their surroundings, which include processes such as proliferation, invasion, metastasis, angiogenesis, and the development of drug resistance [19–21]. The therapeutic potential of targeting sphingolipid metabolism in oncology has garnered increasing attention, with some pharmacological agents demonstrating efficacy by modulating sphingolipid pathways to inhibit tumor growth and survival, marking them as promising candidates for anticancer therapies [22, 23]. Additionally, molecular markers associated with sphingolipid metabolism, such as metabolizing enzymes, have been proposed as novel diagnostic and prognostic indicators for tumors [24, 25]. Given its involvement in tumor progression, patient prognosis, and therapeutic response, including resistance to immunotherapy, sphingolipid metabolism has emerged as a biologically important process with therapeutic and prognostic relevance in cancer [26, 27]. Nevertheless, the precise molecular mechanisms underlying sphingolipid metabolism in ccRCC and its impact on immunotherapy remain inadequately understood, necessitating further investigation to elucidate the specific pathways by which sphingolipid metabolism affects ccRCC and its potential role in modulating immunotherapeutic responses.

Single‐cell RNA–sequencing (scRNA‐seq) technology affords the unprecedented ability to examine gene expression heterogeneity at the single‐cell level, yet the inherent high dimensionality and complexity of scRNA‐seq data present significant analytical challenges [28, 29]. Nonnegative matrix factorization (NMF), an unsupervised machine learning technique, addresses these challenges by effectively reducing data dimensionality and extracting critical features through the decomposition of the high‐dimensional expression matrix into two lower dimensional nonnegative matrices [30–32]. The nonnegativity constraint inherent to NMF enhances its suitability for gene expression data, facilitating dimensionality reduction that renders the data more interpretable and yielding biologically meaningful section‐based representations [33–35]. This property makes NMF particularly suitable for resolving intracell‐type heterogeneity and identifying metagene‐defined subclusters from scRNA‐seq data, thereby enabling the extraction of sphingolipid metabolism–related characteristic genes (SRCGs) in a biologically interpretable manner. In our investigation, NMF was employed to identify distinct cell types, uncover gene modules, and analyze intercellular communication, thereby elucidating the role of sphingolipid metabolism–related genes (SRGs) within the TME of ccRCC.

To advance our understanding of the interplay between sphingolipid metabolism and ccRCC, we aimed to explore specific molecular pathways, cellular communication networks, and the implications for immunotherapy. This inquiry seeks to uncover the mechanistic underpinnings of sphingolipid metabolism in the etiology and progression of ccRCC, with the ultimate goal of informing novel therapeutic strategies and prognostic assessments. Consequently, this study centers on the sphingolipid metabolism–regulated TME, analyzed through single‐cell NMF, and from this foundation, we developed a prognostic scoring model based on SRCGs to predict patient outcomes in ccRCC. By combining single‐cell transcriptomic analysis with NMF, our study provides a refined framework for identifying sphingolipid metabolism–related cellular heterogeneity in the ccRCC microenvironment. We further translated these findings into a prognostic model using 101 machine learning combinations, thereby linking tumor metabolic heterogeneity to patient stratification and potential therapeutic insight.

## 2. Material and Methods

### 2.1. Research Data Acquisition and Processing

The raw datasets utilized in this investigation were sourced from Gene Expression Omnibus (GEO), UCSC Xena, International Cancer Genome Consortium (ICGC), and ArrayExpress. The scRNA‐seq data pertaining to ccRCC patients were exclusively acquired from the GEO database (http://www.ncbi.nlm.nih.gov/geo), encompassing three tumor datasets (GSE152938‐GSM4630028, GSE171306‐GSM5222644, and GSE121638‐GSM3440845) and one normal dataset (GSE152938‐GSM4630031). Bulk RNA‐seq data were retrieved from UCSC Xena and ICGC, corresponding, respectively, to the train and test groups. The raw data underwent annotation using the respective files, with mean values computed for identical probes. For the construction of 101 machine learning models, the training dataset was derived from UCSC Xena, aligning with the train group in bulk RNA‐seq, while the validation dataset originated from ArrayExpress. Before machine learning analysis, expression data from different cohorts were uniformly preprocessed using the “limma” package and subsequently adjusted for batch effects using the “sva” package to improve cross‐cohort comparability and analytical robustness. Somatic mutation data of the TCGA‐KIRC cohort were obtained from UCSC Xena for subsequent mutational analyses. SRGs were identified through the Gene Set Enrichment Analysis (GSEA) database.

### 2.2. Single‐Cell Data Processing and Clustering Analysis for ccRCC

The single‐cell dataset for ccRCC was imported using the Read10X function, and the data were subsequently converted into Seurat objects within the R environment via the “Seurat” package. The three tumor samples were integrated with one normal sample through the merge function. Quality control was conducted by evaluating the proportions of mitochondrial ribosomes and erythrocytes within the cells. Cells with gene counts exceeding 200 but below 4000 were included to exclude those with atypical gene expression levels. Additionally, cells with a mitochondrial RNA proportion above 15% (pctMT = 15) were excluded, as high mitochondrial content indicates poor cell quality, thereby ensuring only high‐quality cells were analyzed. The integrated dataset underwent normalization via the NormalizeData function. For clustering analysis, principal component analysis (PCA) was employed for dimensionality reduction. The FindVariableFeatures function identified the Top 2000 genes with high variability. Batch effects were mitigated using the “harmony” package, and principal component scores from 1 to 10 were applied during the downscaling process. The resulting unsupervised cell clusters were visualized using t‐SNE and UMAP plots. Cell clusters were annotated using marker genes from prior studies, and differential expression of cluster‐specific genes was illustrated with heatmaps, highlighting characteristic genes.

### 2.3. NMF Analysis and Identification of SRCGs

NMF was selected in this study because it enables the decomposition of scRNA‐seq expression matrices into biologically interpretable metagene signatures, which is particularly advantageous for resolving cellular heterogeneity within individual immune/stromal cell types and for identifying SRCG‐related subclusters. Unsupervised NMF analysis was executed using the NMF R software package, beginning with the extraction of data pertinent to each cell cluster. Dimensionality reduction was achieved through the application of NMF, leading to the formation of distinct cell clusters. Visualize these clusters through the UMAP method. Cluster‐specific marker genes were identified using the FindAllMarkers function in Seurat. For each NMF‐derived cluster, marker genes were ranked according to avg_log2FC, and the top‐ranked gene was used as the leading gene for cluster annotation. If the leading gene belonged to the predefined SRG set and simultaneously met the criteria of adjusted *p* < 0.05 and avg_log2FC > 1, the cluster was designated as an SRG‐related cluster and named in the format of “Gene–CellType–Cx”; in this case, the leading gene was defined as the metagene. NMF clusters were annotated according to their leading metagene for naming convenience; however, the prognostic or biological behavior of a given cluster reflects the overall transcriptional characteristics of that cell subset rather than the isolated effect of the naming gene alone. If the leading gene belonged to the SRG set but failed to meet either of the above thresholds, the cluster was labeled as “Unclear.” Clusters whose leading gene was not included in the SRG set were labeled as “Non‐SL.” Clusters labeled as “Unclear” or “Non‐SL” were retained for descriptive presentation of NMF‐derived heterogeneity but were excluded from downstream SRCG extraction and prognostic model construction.

### 2.4. SCENIC Analysis of Transcription Factor (TF) Activity

To assess TF regulatory activity across NMF‐defined clusters, SCENIC analysis was performed on raw RNA count matrices. For each analyzed cell subset, 200 cells were randomly sampled to reduce computational burden. SCENIC was initialized with the human setting (org =  ^“^hgnc^”^), using motifAnnotations_hgnc_v9 and the hg19‐tss‐centered‐10 kb‐7species.mc9nr.feather database. Genes were filtered according to minCountsPerGene = 3 × 0.1 × nCells and minSamples = 0.1 × nCells. Regulatory networks were inferred by correlation analysis and GENIE3, and regulons were generated with coexMethod =  ^“^top50^”^. Regulon activity was quantified by AUCell, followed by binarization. Cluster‐level average regulon activity was subsequently visualized as a heatmap.

### 2.5. Prognostic Model Construction and Evaluation Using Machine Learning

We employed a suite of 10 algorithms to perform variable screening and construct a prognostic model. Model training utilized expression profile data and survival outcomes from the training set, with each trained model subsequently evaluated against the validation set. The concordance index (*C*‐index) was computed across all cohorts to gauge the predictive accuracy of the models. Differences in performance among algorithms were visualized through heatmaps, with darker shades signifying higher accuracy and better model performance. Ultimately, a model was constructed based on the Lasso+SuperPC combination. The risk score for each patient was calculated as the weighted sum of the expression levels of the 12 SRCGs using the coefficients derived from the final Lasso+SuperPC model. The corresponding coefficients are provided in the Result section. Survival curves for both the training and validation sets were then plotted, and the predictive efficacy of various clinical features for 1‐, 3‐, and 5‐year survival was assessed using the “survival,” “survminer,” and “timeROC” R packages. Box plots of clinical features were generated using the “ggpubr” package for further comparison.

### 2.6. Development and Validation of the Prognostic Nomogram

A predictive nomogram was developed utilizing risk scores, followed by internal validation through calibration plots to verify the model′s accuracy. Additionally, the nomogram′s predictive capability was evaluated by plotting ROC curves; employing the “survival,” “survminer,” and “timeROC” R packages; and comparing its performance against other variables.

### 2.7. Immune Cell Profiling and Immune Function Analysis

We employed CIBERSORT to assess the relative proportions of immune cells within the TME, contrasting the cellular composition between the low‐ and high‐risk groups. The differential expression of immune cells across these groups was visualized accordingly. Furthermore, the “GSVA” R package was utilized to calculate ssGSEA scores, providing immune function scores and illustrating regulatory changes in immune function, thereby facilitating a deeper exploration of immune correlations.

### 2.8. Pathway and Functional Enrichment Analysis of SRCGs

To elucidate the potential pathways and functional roles of the modeled SRCGs, we conducted Gene Ontology (GO) enrichment and KEGG pathway analyses using the “clusterProfiler” R package. The GO terms were classified into biological processes (BPs), cellular components (CCs), and molecular functions (MFs), with significance thresholds set at a *p* value and *q*‐value of 0.05. We focused on the Top 10 GO‐enriched pathways and the Top 12 KEGG pathways exhibiting the highest enrichment significance, presenting detailed insights into the most markedly enriched pathways.

### 2.9. Drug Sensitivity Analysis

Drug sensitivity analysis is an approach employed to elucidate potential therapeutic targets by examining the correlation between gene expression profiles and drug responses. Utilizing the R software package “OncoPredict,” we evaluated the sensitivity of ccRCC patients to various standard chemotherapeutic agents, informed by 12 modeled SRCGs. This analysis not only establishes a theoretical framework for personalized clinical interventions but also identifies prospective drug sensitivity biomarkers, thereby aiding in the refinement of therapeutic strategies for tumor patients.

### 2.10. Protein–Protein Interaction (PPI) Network Construction and Analysis

PPI analysis of the modeled SRCGs was performed using the STRING database (https://string-db.org). Genes were entered using the “Multiple proteins” option, with *Homo sapiens* specified as the reference organism. The minimum required interaction score was set to 0.400 (medium confidence) for network construction.

### 2.11. Mutation Analysis

Somatic mutation analysis was performed for TCGA‐KIRC samples. The overall mutation landscape was obtained using the TCGAmutations package and visualized with maftools after constructing a mutation annotation format (MAF) object with matched clinical information. Frequently mutated genes were displayed using oncoplots. For mutation burden comparison, somatic mutation data from KIRC were integrated with the risk grouping information of the TCGA training cohort. Total mutation count was defined as the number of all mutation events per sample, whereas nonsynonymous mutation count was calculated based on protein‐altering variant types. Differences in mutation burden between the high‐ and low‐risk groups were evaluated using the Wilcoxon rank‐sum test. All analyses were conducted in R with the TCGAmutations, maftools, data.table, dplyr, and ggplot2 packages.

### 2.12. Statistical Analysis

Data were subjected to processing and statistical evaluation utilizing R software Version 4.3.1. Significance was assessed with *p* values and false discovery rates (FDRs) set at < 0.05. All analyses were performed in R. The major R packages used in this study included Seurat (v5.4.0) for single‐cell analysis, NMF (v0.28) for matrix factorization, harmony (v1.2.4) for batch correction, and limma (v3.62.2) and sva (v3.54.0) for bulk transcriptomic data processing.

## 3. Result

### 3.1. Distribution of SRGs in TME Cells in ccRCC

**Figure 1:**
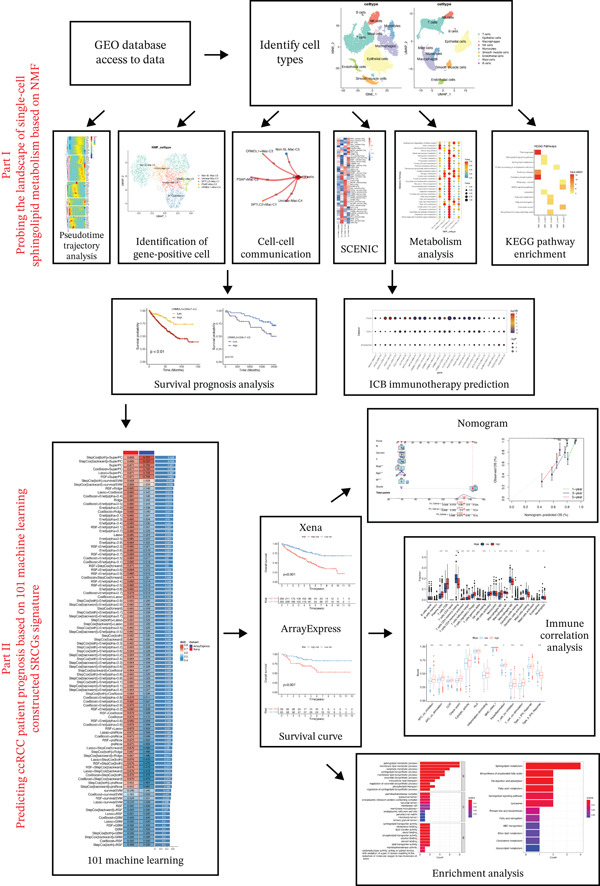
Research process flowchart.

**Figure 2:**
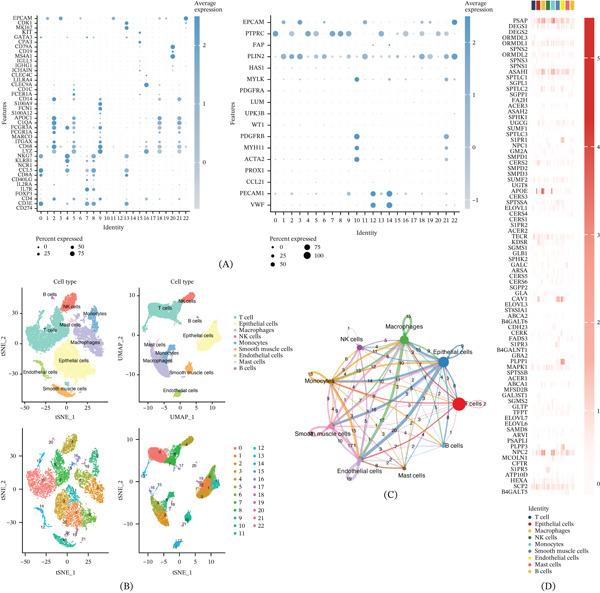
Distribution and communication of SRGs in TME cells of ccRCC. (A) Identification of 23 distinct clusters. (B) Annotation of the clusters revealed nine major cell types. (C) Intercellular communication analysis. (D) Visualization of SRG expression patterns across the identified cell types in ccRCC samples.

The research workflow of the study is outlined in the graphical flowchart (Figure 1), which visually represents the key steps, from dataset acquisition and single‐cell clustering to the construction and evaluation of the prognostic model using 101 machine learning combinations. In this investigation, we analyzed three tumor datasets (GSE152938‐GSM4630028, GSE171306‐GSM5222644, and GSE121638‐GSM3440845) alongside a normal dataset (GSE152938‐GSM4630031) to elucidate the role of SRGs in ccRCC. After quality control of the scRNA‐seq data, batch effects were mitigated using the Harmony package, leading to the identification of 23 distinct clusters. Annotating these clusters (Figure 2A) revealed nine distinct cell types: T cells, epithelial cells, macrophages, NK cells, monocytes, smooth muscle cells, endothelial cells, mast cells, and B cells (Figure 2B). Subsequent analysis of intercellular communication across these cell types (Figure 2C) highlighted robust interactions among epithelial cells, macrophages, monocytes, endothelial cells, and smooth muscle cells, while T cells, NK cells, mast cells, and B cells demonstrated comparatively weaker communication. Finally, we visualized the expression patterns of SRGs across the identified cell types in ccRCC samples (Figure 2D).

### 3.2. Immune Cell Dynamics and Sphingolipid Gene Regulation in NK Cells of ccRCC

**Figure 3:**
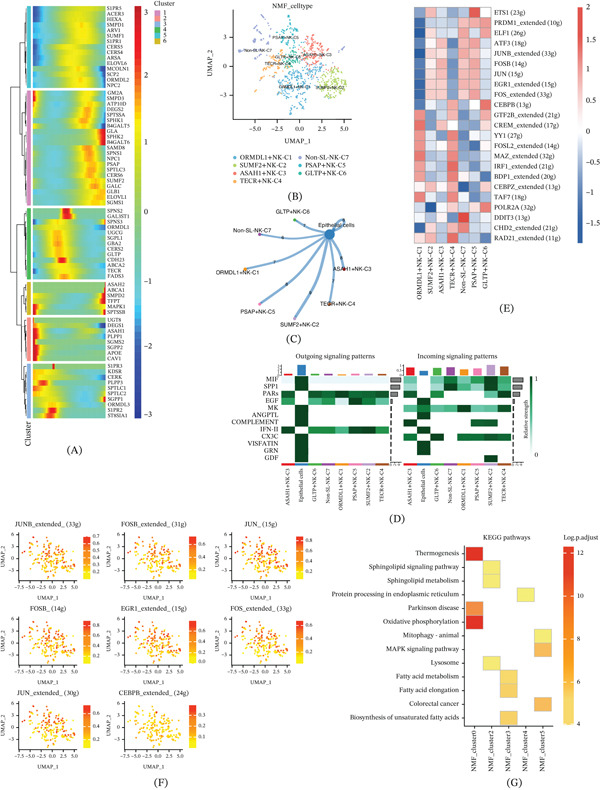
NK cells of ccRCC. (A) Temporal changes in SRG expression during NK‐cell development. (B) Identification of seven NK‐cell NMF clusters. (C) Intercellular communication analysis between NK‐cell clusters and epithelial cells. (D) Outgoing and incoming signaling patterns. (E) Transcription factor (TF) activity analysis. (F) Visualization of the expression patterns of eight TFs in NK cells. (G) KEGG enrichment analysis.

As a critical component of the immune system, NK cells are instrumental in recognizing and eliminating infected and tumor cells. In this study, we isolated 1132 NK cells from scRNA‐seq data to assess the impact of SRGs on their function in ccRCC. Through a proposed time‐trajectory analysis, we identified a progressive upregulation of multiple SRGs, including GLA, SPHK2, and B4GALT6, during NK‐cell development. Conversely, genes such as ORMDL1, ASAH1, PLPP1, and APOE exhibited significant downregulation over time, highlighting distinct temporal gene expression patterns (Figure 3A).To deepen the understanding of the association between SRGs and NK cells, we identified six distinct NK‐cell NMF clusters annotated by representative SRG metagenes, designated as ORMDL1+NK‐C1, SUMF2+NK‐C2, ASAH1+NK‐C3, TECR+NK‐C4, PSAP+NK‐C5, and GLTP+NK‐C6. Additionally, a cluster of non‐SRG, termed Non‐SL‐NK‐C7, was identified (Figure 3B). Analysis of intercellular communication between these NK‐cell clusters and epithelial cells revealed robust interaction across all clusters, with the SUMF2‐mediated NK‐cell cluster exhibiting the highest communication strength (Figure 3C). Further, we examined efferent and afferent signaling patterns between these clusters and epithelial cells. Signaling molecules such as migration inhibitory factor (MIF), SPP1, and PARs were notably activated, with epithelial cells predominating in efferent signaling, whereas NK‐cell clusters exhibited stronger afferent signaling (Figure 3D). In the SCENIC analysis, significant heterogeneity was observed in TF activity among the NK‐cell clusters: ORMDL1+NK‐C1 and TECR+NK‐C4 showed lower activity of JUNB_extended and JUN, while SUMF2+NK‐C2, ASAH1+NK‐C3, and PSAP+NK‐C5 had heightened activity. Conversely, ORMDL1+NK‐C1 and TECR+NK‐C4 exhibited higher activity of MAZ_extended and IRF1_extended, with ORMDL1+NK‐C1 demonstrating the highest TF activity (Figure 3E). The expression of eight TFs in NK cells was also visualized (Figure 3F). Finally, KEGG functional enrichment analysis of the NK‐cell clusters revealed significant enrichment of pathways related to thermogenesis and oxidative phosphorylation in NMF_cluster0 (ORMDL1+NK‐C1) (Figure 3G), suggesting that this subset may exhibit a metabolically active state relative to the other NK‐cell clusters.

### 3.3. SRG Expression and Functional Dynamics in T Cells

**Figure 4:**
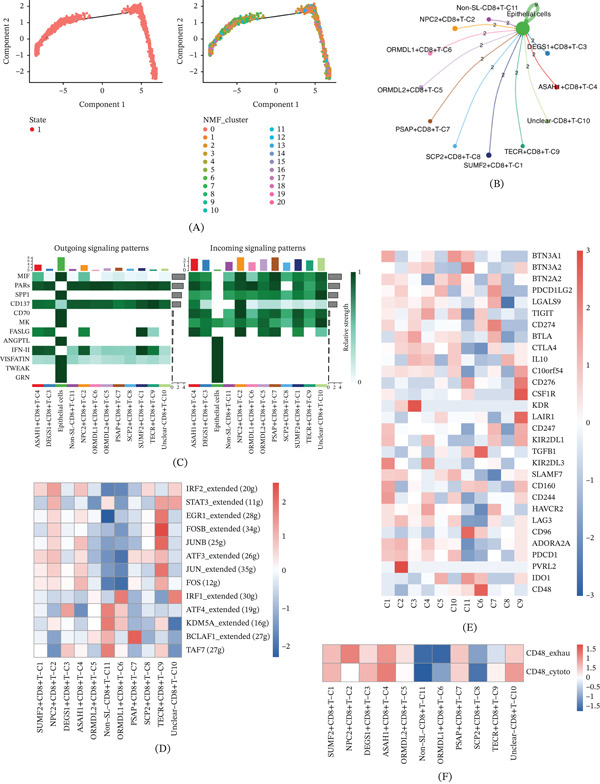
T cells of ccRCC. (A) Time trajectory analysis showing dynamic changes during CD8+ T‐cell maturation. (B) Intercellular communication analysis between T‐cell clusters and epithelial cells. (C) Outgoing and incoming signaling patterns. (D) TF activity analysis. (E) Expression of immunosuppressive factors across NMF clusters. (F) T‐cell function scores, derived from CD8+ T‐cell exhaustion and cytotoxicity markers.

T cells, integral to the immune system, are crucial for defending against infections and tumors. From the scRNA‐seq data, we identified 5304 T cells and subsequently isolated CD8+ T cells. Through time trajectory analysis, it was determined that CD8+ T cells possess a singular developmental state (State 1), with their subpopulations dispersed across the developmental timeline, indicating dynamic changes during CD8+ T‐cell maturation (Figure 4A). NMF clustering analysis identified nine distinct clusters: SUMF2+CD8+T‐C1, NPC2+CD8+T‐C2, DEGS1+CD8+T‐C3, ASAH1+CD8+T‐C4, ORMDL2+CD8+T‐C5, ORMDL1+CD8+T‐C6, PSAP+CD8+T‐C7, SCP2+CD8+T‐C8, and TECR+CD8+T‐C9. Additionally, an Unclear‐CD8+T‐C10 cluster and a non‐SRG cluster (Non‐SL‐CD8+T‐C11) were identified. Subsequent analysis of intercellular communication revealed relatively uniform interaction intensity between the nine T‐cell clusters and epithelial cells (Figure 4B), indicating limited heterogeneity in their epithelial communication patterns. In both efferent and afferent signaling, analogous to the NK‐cell clusters, T‐cell clusters exhibited activation of signaling pathways mediated by molecules such as MIF, PARs, and SPP1. Efferent signaling was predominantly higher in epithelial cells, while afferent signaling was more pronounced in T‐cell clusters (Figure 4C). SCENIC analysis revealed considerable heterogeneity among the T‐cell clusters (Figure 4D), indicating that these subsets may be governed by distinct transcriptional regulatory programs. The expression of immunosuppressive factors was also evaluated across the NMF clusters, revealing distinct profiles, with SUMF2+CD8+T‐C1 exhibiting elevated expression levels (Figure 4E), suggesting that this subset may be more closely associated with an immunosuppressive state. Finally, T‐cell function scores, derived from markers associated with CD8+ T‐cell exhaustion and cytotoxic T cells, indicated that ASAH1+CD8+T‐C4 had higher scores in both T‐cell exhaustion and cytotoxicity, whereas ORMDL1+CD8+T‐C6 and SCP2+CD8+T‐C8 scored lower (Figure 4F), suggesting functional heterogeneity among the T‐cell subsets, with ASAH1+CD8+T‐C4 representing a relatively more activated yet exhausted state. These findings underscore the significant functional heterogeneity among the NMF clusters.

### 3.4. Macrophages Regulated by SRGs in ccRCC

**Figure 5:**
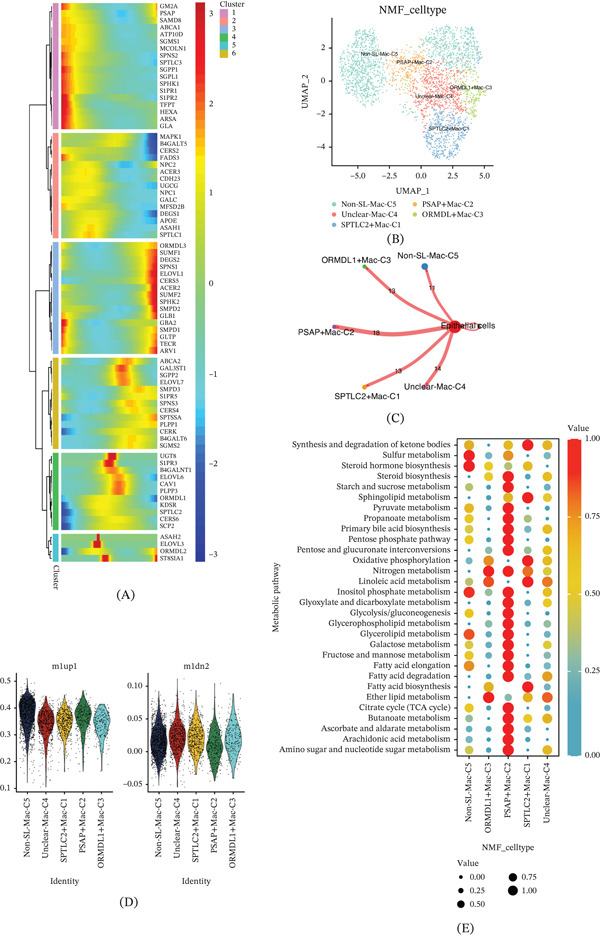
Macrophages of ccRCC. (A) Temporal changes in SRG expression during macrophage development. (B) Identification of five macrophage NMF clusters. (C) Intercellular communication analysis between macrophage clusters and epithelial cells. (D) Scoring of M1 and M2 macrophage subtypes. (E) Analysis of metabolic pathway enrichment within macrophage clusters.

Macrophages, with their fundamental roles in immunoregulation, phagocytosis, and antigen presentation, are pivotal in the body′s immune defense, self‐regulation, and surveillance. From scRNA‐seq data, we isolated 2892 macrophages and, through the proposed time trajectory analysis, observed that the expression of most SRGs in macrophages increased during the mid‐to‐late stages, with genes such as SUMF2 and DEGS2 being upregulated, whereas genes like SPTLC3 and DEGS1 showed significant downregulation in the late stage (Figure 5A).NMF analysis identified five distinct cell clusters, including three SRG‐defined clusters: SPTLC2+Mac‐C1, PSAP+Mac‐C2, and ORMDL1+Mac‐C3, along with an Unclear‐Mac‐C4 and a Non‐SL‐Mac‐C5 cluster (Figure 5B). Intercellular communication analysis revealed robust interaction between all five macrophage clusters and epithelial cells, with the PSAP+Mac‐C2 cluster exhibiting the strongest communication (Figure 5C). Subsequent scoring of the M1 and M2 macrophage subtypes showed that among the SRG‐defined clusters, PSAP+Mac‐C2 had higher scores in M1‐type (proinflammatory) macrophages, while SPTLC2+Mac‐C1 was more associated with M2‐type (anti‐inflammatory) macrophages. Notably, all macrophage clusters exhibited higher scores for M1 macrophages (Figure 5D). Finally, an analysis of metabolic pathway enrichment within macrophage clusters revealed that the PSAP+Mac‐C2 cluster had the most significant enrichment in pathways related to steroid biosynthesis, starch and sucrose metabolism, and the citrate cycle (TCA cycle) (Figure 5E).

### 3.5. SRGs Mediate the Functions of Monocytes, Mast Cells, Endothelial Cells, and Smooth Muscle Cells in ccRCC

**Figure 6:**
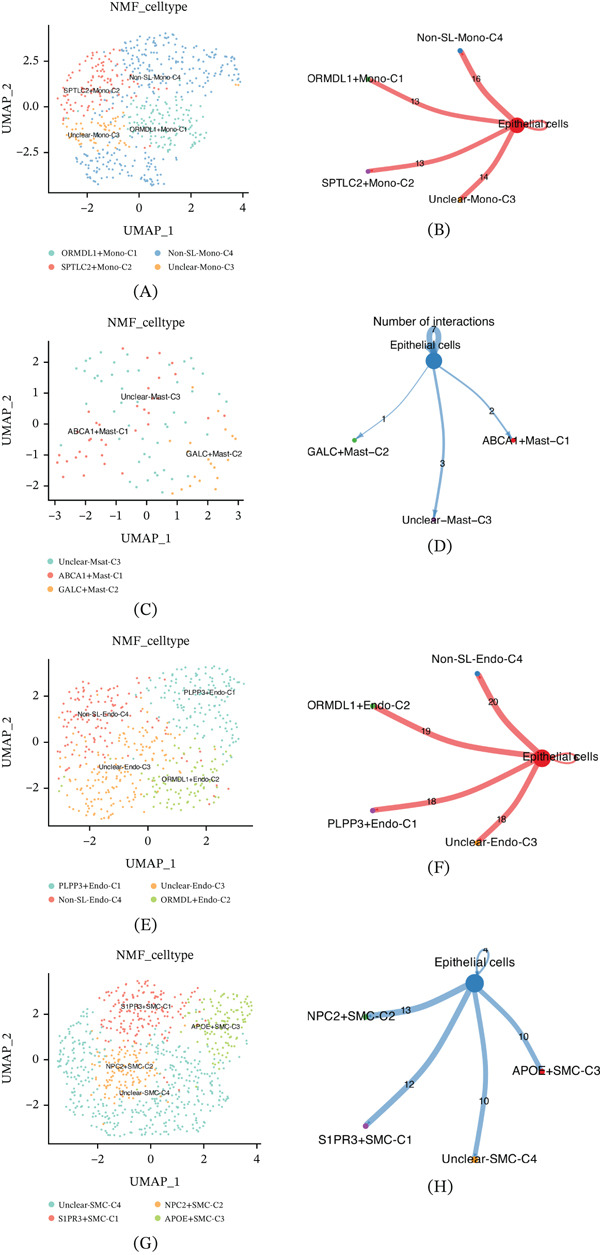
Monocytes, mast cells, endothelial cells, and smooth muscle cells of ccRCC. (A) Identification of four monocyte NMF clusters. (B) Intercellular communication analysis between monocyte clusters and epithelial cells. (C) Identification of three mast cell NMF clusters. (D) Intercellular communication analysis between mast cell clusters and epithelial cells. (E) Identification of four endothelial cell NMF clusters. (F) Intercellular communication analysis between endothelial cell clusters and epithelial cells. (G) Identification of four smooth muscle cell NMF clusters. (H) Intercellular communication analysis between smooth muscle cell clusters and epithelial cells.

Monocytes exert significant regulatory influence in oncogenesis, particularly through modulation of tumor growth and metastasis. From the scRNA‐seq analysis, 835 monocytes were isolated, and subsequent NMF‐based clustering revealed four distinct subpopulations: ORMDL1+Mono‐C1, SPTLC2+Mono‐C2, Unclear‐Mono‐C3, and Non‐SL‐Mono‐C4 (Figure 6A). Each monocyte cluster demonstrated a robust cellular communication pattern with epithelial cells (Figure 6B).

In the context of the TME, mast cells—key players in inflammation and immune response—contribute notably to tumorigenesis. A total of 196 mast cells were identified and, through NMF‐based clustering, categorized into three distinct clusters: ABCA1+Mast‐C1, GALC+Mast‐C2, and Unclear‐Mast‐C3 (Figure 6C). However, cellular communication analysis revealed that these mast cell clusters exhibited relatively weak communication with epithelial cells (Figure 6D).

Endothelial cells, which play a critical role in angiogenesis by modulating blood supply and nutrient delivery, thereby influencing tumor growth, spread, and metastasis, were stratified into four clusters: PLPP3+Endo‐C1, ORMDL1+Endo‐C2, Unclear‐Endo‐C3, and Non‐SL‐Endo‐C4 (Figure 6E). Unlike mast cells, all endothelial cell clusters displayed strong communicative interactions with epithelial cells (Figure 6F).

Smooth muscle cells, implicated in the regulation of growth, invasion, and metastasis in ccRCC, were similarly clustered into four subpopulations via NMF analysis: S1PR3+SMC‐C1, NPC2+SMC‐C2, APOE+SMC‐C3, and Unclear‐SMC‐C4 (Figure 6G). Cellular communication analysis further indicated that all four smooth muscle cell clusters exhibited potent communication with epithelial cells, with NPC2+SMC‐C2 demonstrating the most pronounced interaction (Figure 6H).

### 3.6. Prognostic Implications of SRG‐Mediated NMF Clusters in ccRCC

**Figure 7:**
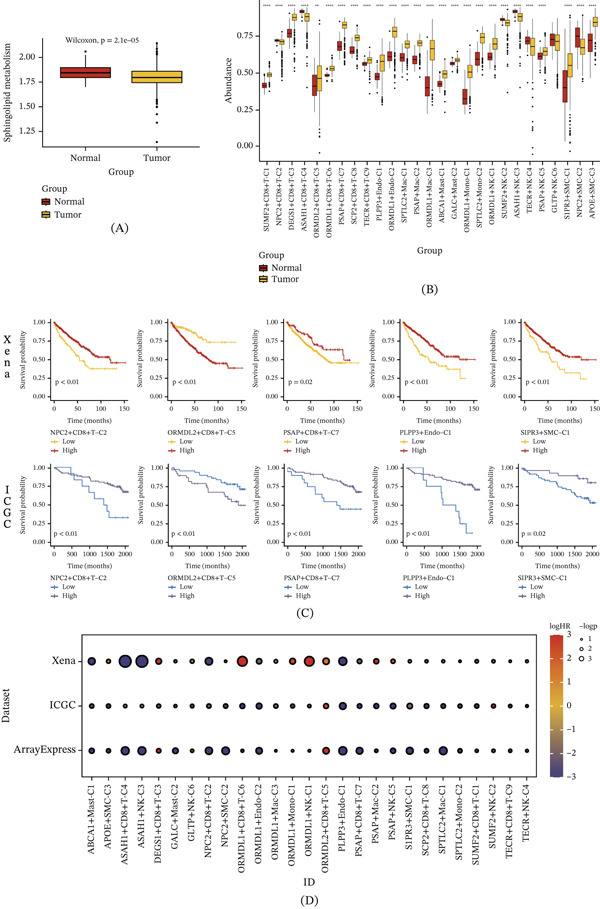
Prognostic implications of SRG‐mediated NMF clusters in ccRCC. (A) Comparison of SRG expression in normal and tumor samples at the bulk level. (B) Differential enrichment of SRG in NMF cell clusters. (C) Kaplan–Meier survival analysis for various NMF clusters. (D) Validation of prognostic findings across the Xena, ICGC, and ArrayExpress datasets.

To further investigate the impact of SRG‐mediated cell clusters identified through scRNA‐seq in ccRCC, data from the Xena database, the ICGC bulk RNA‐seq dataset, and individual survival data were integrated. Marker genes specific to each NMF cluster were utilized to score the respective clusters within the bulk RNA‐seq samples, forming the basis for subsequent analyses. Initial examination of these scores across normal and tumor samples at the bulk level revealed significantly higher overall SRG expression in the normal group than in the tumor group (Figure 7A). In contrast, analysis of NMF cluster–derived signature scores showed that most metagene‐defined clusters were more enriched in the tumor group (Figure 7B). Subsequent Kaplan–Meier survival analysis of each cell cluster in ccRCC identified several NMF clusters with significant prognostic relevance across the Xena and ICGC datasets, including NPC2+CD8+T‐C2, ORMDL2+CD8+T‐C5, PSAP+CD8+T‐C7, PLPP3+Endo‐C1, and S1PR3+SMC‐C1. Among these, ORMDL2+CD8+T‐C5 was associated with poor prognosis, whereas the remaining clusters were linked to more favorable outcomes (Figure 7C). These prognostic patterns were further corroborated in the ArrayExpress dataset, supporting the robustness of the identified clusters across independent cohorts (Figure 7D). It should be noted that the prognostic direction of a metagene‐defined cluster does not necessarily reflect the prognostic role of the naming gene itself, as each cluster represents a broader transcriptional program rather than the effect of a single gene.

### 3.7. Influence of SRG‐Associated NMF Clusters on ccRCC Immunotherapy and Validation in a Bladder Cancer Cohort

**Figure 8:**
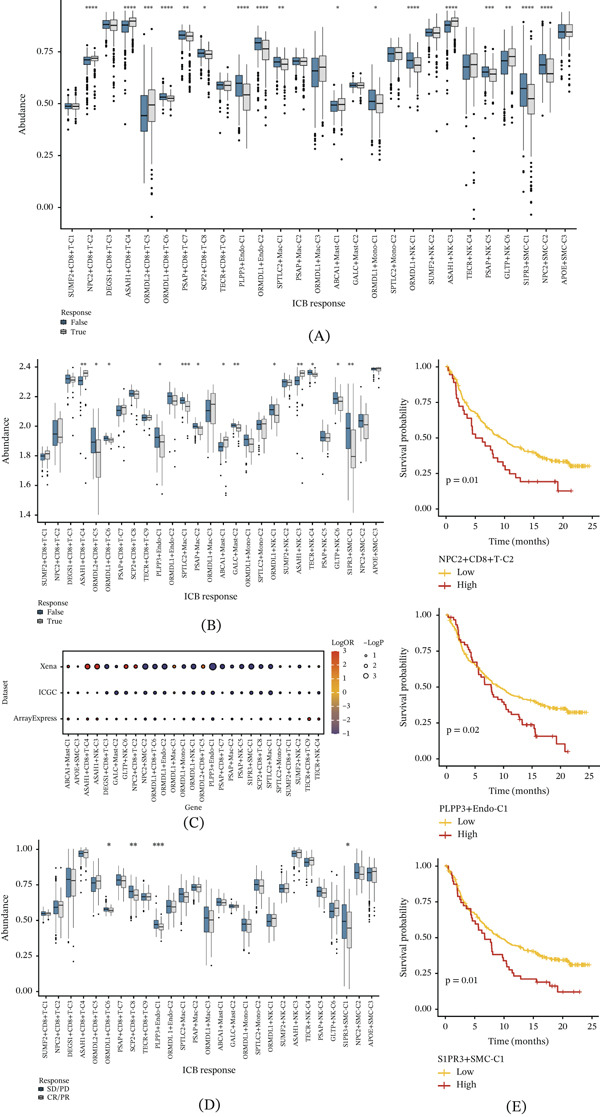
Influence of NMF clusters on predicted immunotherapy response in ccRCC and validation in a bladder cancer cohort. (A) Comparison of NMF cluster scores between predicted responders and predicted nonresponders in the Xena dataset. (B) Comparison of NMF cluster scores between predicted responders and predicted nonresponders in the ICGC dataset. (C) Logistic regression analysis of the association between NMF cluster scores and predicted immunotherapy response. (D) Comparison of NMF cluster scores between the SD/PD and CR/PR groups in the bladder cancer immunotherapy cohort. (E) Survival analysis for selected clusters in the bladder cancer cohort.

To further explore the immunotherapeutic implications of the identified NMF clusters, we integrated predicted immunotherapy response classifications with the NMF cluster scores for each sample. In the Xena cohort, PLPP3+Endo‐C1, ORMDL1+Endo‐C2, and ORMDL1+NK‐C1 showed significantly higher scores in the predicted nonresponder group than in the predicted responder group (Figure 8A). Similarly, in the ICGC cohort, PLPP3+Endo‐C1, ORMDL1+CD8+T‐C6, and S1PR3+SMC‐C1 also exhibited higher scores in the predicted nonresponder group (Figure 8B). These findings suggest that specific cell‐related NMF clusters may be associated with less favorable predicted immunotherapy response in ccRCC. Furthermore, logistic regression analysis showed negative logOR values for these NMF clusters (Figure 8C), further supporting their association with predicted nonresponder status in ccRCC. To substantiate our findings, we conducted additional analyses utilizing a real‐world cancer immunotherapy cohort. We observed that SCP2+CD8+T‐C8, PLPP3+Endo‐C1, and S1PR3+SMC‐C1 clusters exhibited significantly higher NMF scores in the SD/PD (stable disease/progressive disease) group than in the CR/PR (complete response/partial response) group concerning immunotherapy response, further supporting their association with less favorable immunotherapy outcomes. (Figure 8D). In addition, survival analysis in the bladder cancer cohort showed that NPC2+CD8+T‐C2, PLPP3+Endo‐C1, and S1PR3+SMC‐C1 were associated with poor prognosis, whereas these same clusters were linked to favorable prognosis in ccRCC (Figure 8E). This discrepancy suggests that the prognostic effects of similar cell subsets may be context‐dependent and influenced by differences in the TME across cancer types.

### 3.8. Construction and Validation of 101 Combined Machine Learning Algorithm Screening Models

**Figure 9:**
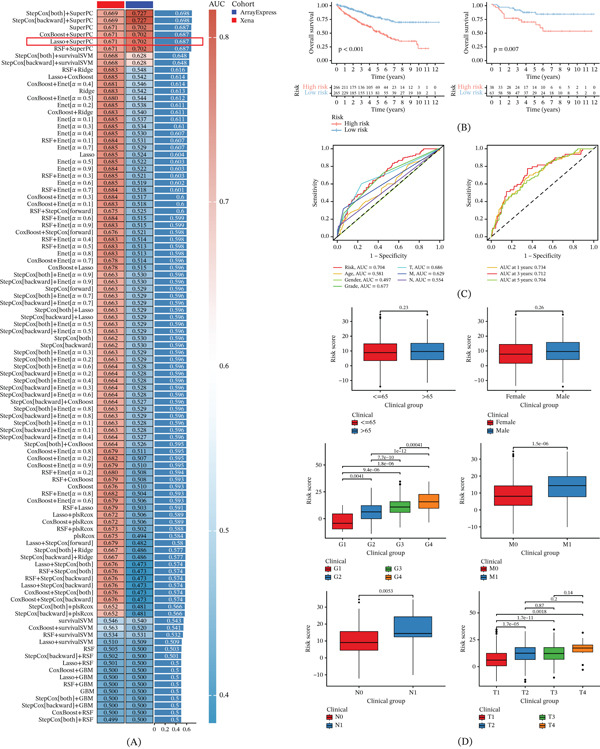
Construction and validation of 101 combined machine learning algorithm screening models. (A) 101 machine learning algorithms and the best selected Lasso+SuperPC combination model. (B) Survival analysis of the high‐ and low‐risk groups based on the SRCGPS. (C) ROC curves for clinical variables and SRCGPS. (D) Clinical correlation analysis of SRCGPS‐associated risk scores.

To ensure consistency in downstream modeling, only metagenes from SRG‐defined NMF clusters were included as SRCGs for the 101 machine learning framework; clusters annotated as “Unclear” or “Non‐SL” were not entered into prognostic model construction. Multiple machine learning algorithms were employed to develop and validate a robust prognostic model, analyzing 16 metagenes derived from NMF cluster analysis. The UCSC Xena dataset was used for training, while the ArrayExpress dataset served for validation. Following effect correction to ensure analytical precision, we applied 10 distinct machine learning algorithms within a 10‐fold cross‐validation framework, generating 101 combinations to construct the sphingolipid metabolism–related characteristic gene prognostic signature (SRCGPS). The model′s performance was assessed using the *C*‐index, with models achieving an average *C* − index > 0.65 being further evaluated for predictive ability in both training and validation sets. Ultimately, the Lasso+SuperPC combination model was selected as the optimal model (Figure 9A), comprising 12 SRCGs with the following coefficients: SUMF2 (1.151140), APOE (2.596089), ORMDL1 (3.168305), ORMDL2 (1.039322), TECR (0.978246), DEGS1 (2.176478), GLTP (1.244283), ABCA1 (−0.703668), SCP2 (−1.646555), ASAH1 (−2.532338), GALC (−1.761186), and PLPP3 (−4.380193). Positive coefficients indicated risk‐associated contributions, whereas negative coefficients indicated protective contributions within the risk‐score model. Risk scores were then calculated for each patient and used to divide patients into high‐ and low‐risk groups according to the median score in the training set. Survival analysis confirmed statistically significant prognostic differences between the training and validation sets (*p* < 0.05) (Figure 9B). ROC curves were generated for clinical factors such as age, gender, grade, and staging (T, M, and N), with the area under the curve (AUC) showing that SRCGPS surpassed other clinical variables (AUC = 0.704). The 1‐, 3‐, and 5‐year ROC curves further demonstrated high sensitivity and specificity (AUC = 0.734, 0.712, and 0.704), underscoring the strong discriminative capacity of SRCGPS (Figure 9C). A comprehensive analysis affirmed the Lasso+SuperPC model as a highly accurate predictive tool. Finally, clinical correlation analysis revealed significant differences in SRCGPS‐associated risk across various clinical traits within the TCGA‐KIRC dataset, with the exception of comparisons among age, T2 and T3, T2 and T4, and T3 and T4 stages, which did not exhibit significant differences (*p* < 0.05) (Figure 9D).

### 3.9. Development of Nomogram and Clinical Utility of the Model

**Figure 10:**
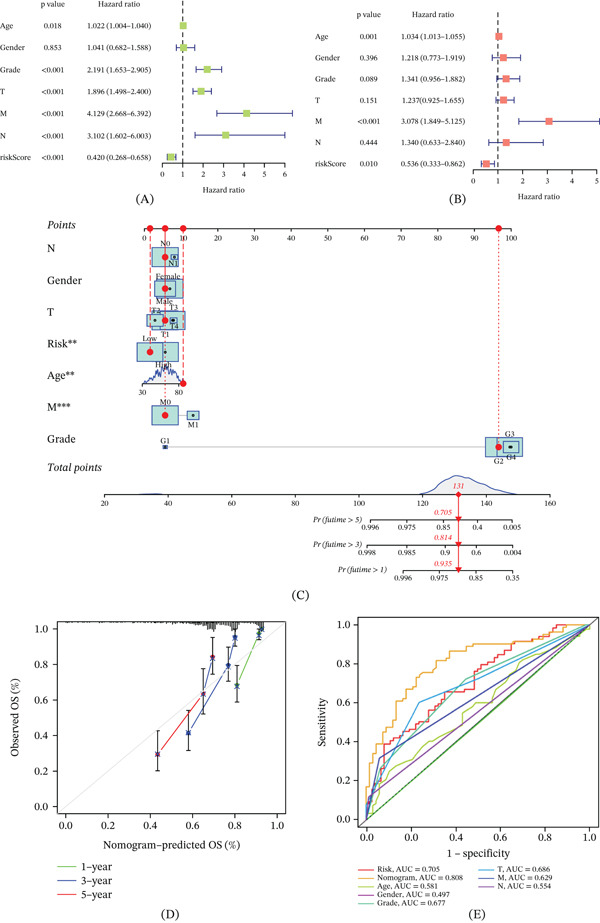
Development of a nomogram. (A) Univariate Cox analysis of prognostic factors. (B) Multivariate Cox analysis identifying independent predictors. (C) Nomogram for predicting 1‐, 3‐, and 5‐year survival. (D) Calibration curves comparing predicted and observed survival. (E) ROC curves for nomogram and clinical factors.

In light of the strong correlations between the constructed risk models and adverse prognosis, forest plots were generated through both univariate and multivariate Cox analyses, integrating overall survival (OS) data of ccRCC patients with their clinical characteristics to assess whether the prognostic features derived from the 12 SRCGs serve as independent survival predictors. Univariate Cox analysis revealed that, with the exception of gender, all factors—riskScore, age, grade, and T, M, and N staging—were significantly linked to ccRCC prognosis (*p* < 0.05) (Figure 10A). Furthermore, multivariate Cox analysis identified riskScore (*p* = 0.010), along with age and M staging, as independent and robust predictors of outcomes, independent of other variables (Figure 10B). To enhance the clinical applicability and utility of the constructed risk model and provide a visual tool for prognostic decision‐making, a nomogram was developed incorporating age, gender, grade, M stage, N stage, T stage, and risk, aimed at predicting 1‐, 3‐, and 5‐year survival probabilities for ccRCC patients (Figure 10C). The consistency between predicted and observed values on the calibration curves for these time points underscores the robust predictive capacity of the nomogram (Figure 10D). Additionally, ROC curves were plotted for risk, nomogram, age, gender, grade, and T, M, and N staging, with the nomogram demonstrating superior predictive accuracy (AUC = 0.808) in comparison to risk (AUC = 0.705) and other clinical features (Figure 10E).

### 3.10. Immune Cell Infiltration Prediction and Immune Function Evaluation

**Figure 11:**
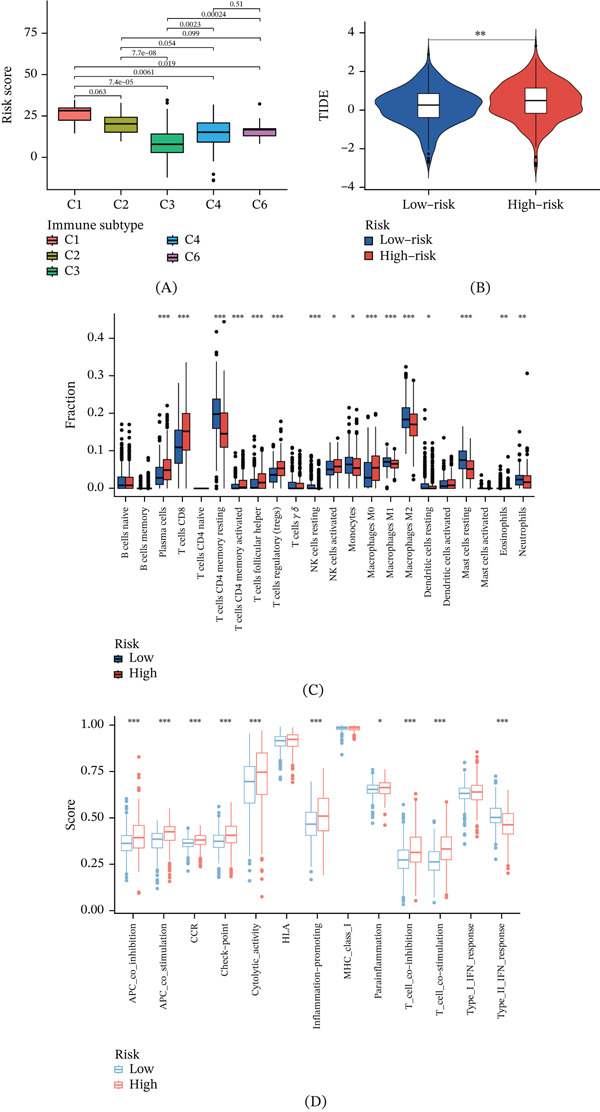
Immunological landscape in ccRCC. (A) Immune risk differences among immune subtypes. (B) TIDE score analysis. (C) Immune cell infiltration variations. (D) Immune function differences.

The pathogenesis of ccRCC is intricately linked to alterations in the immune system. To explore these associations, we initially conducted immunophenotyping analysis, which elucidates the tumor′s immune landscape and informs the development of clinical interventions. Notably, substantial differences were observed among C1, C3, C4, and C6, with C1 presenting the highest risk and C3 the lowest (Figure 11A). TIDE score analysis revealed considerable contrasts between the high‐ and low‐risk groups, highlighting a greater susceptibility to immune escape in the high‐risk group, along with poorer responses to immunotherapy (Figure 11B). To substantiate the potential of model genes in forecasting immune cell infiltration, we used the CIBERSORT algorithm, uncovering significant variations in immune cell infiltration across risk groups. Specifically, the low‐risk group demonstrated elevated infiltration of CD4 memory resting T cells, resting NK cells, monocytes, and M1/M2 macrophages. Conversely, the high‐risk group showed increased infiltration of plasma cells, CD8 T cells, regulatory T cells (Tregs), and M0 macrophages (Figure 11C). Beyond cellular infiltration, we also examined disparities in immune function, finding that the high‐risk group scored higher in APC coinhibition, inflammation promotion, and T‐cell coinhibition relative to the low‐risk group (Figure 11D).

### 3.11. Predictive Targeted Drug Sensitivity Analysis

**Figure 12:**
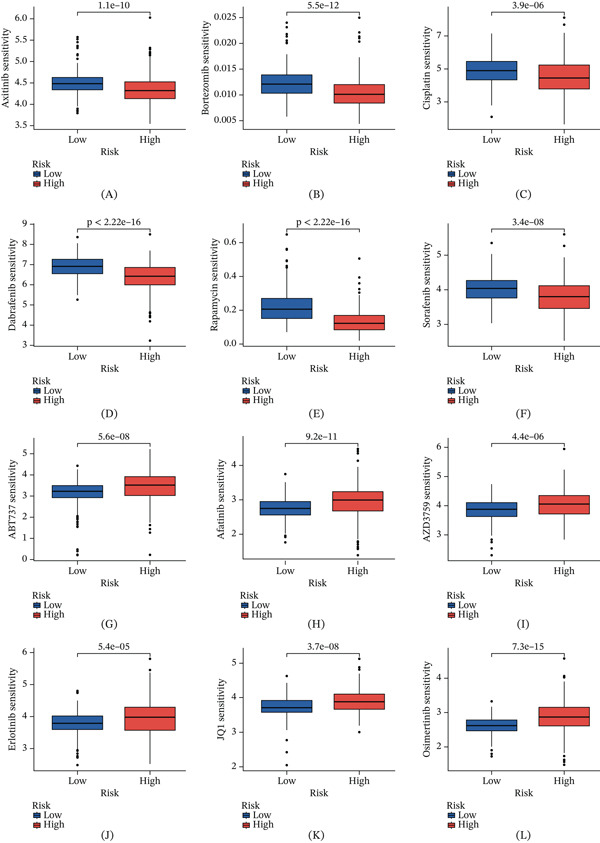
Targeted drug sensitivity in ccRCC. (A–F) Drugs with greater sensitivity to high‐risk groups. (G–L) Drugs with greater sensitivity to low‐risk groups.

Leveraging risk scores and subgroups derived from SRCGs, we performed a differential analysis of predicted drug sensitivity in ccRCC patients to explore potential therapeutic stratification. Our analysis revealed marked differences in estimated drug sensitivity between the high‐risk and low‐risk cohorts (*p* < 0.001). Specifically, the high‐risk group exhibited lower predicted IC_50_ values for axitinib, bortezomib, cisplatin, dabrafenib, rapamycin, and sorafenib (Figures 12A, 12B, 12C, 12D, 12E, and 12F), whereas the low‐risk group showed lower predicted IC_50_ values for ABT737, afatinib, AZD3759, erlotinib, JQ1, and osimertinib (Figures 12G, 12H, 12I, 12J, 12K, and 12L). These findings suggest that the SRCGPS‐defined risk groups may have distinct therapeutic vulnerabilities and that risk stratification could potentially assist in prioritizing candidate agents for different patient subsets.

### 3.12. Enrichment Analysis and Protein Interaction Network of SRCGs

**Figure 13:**
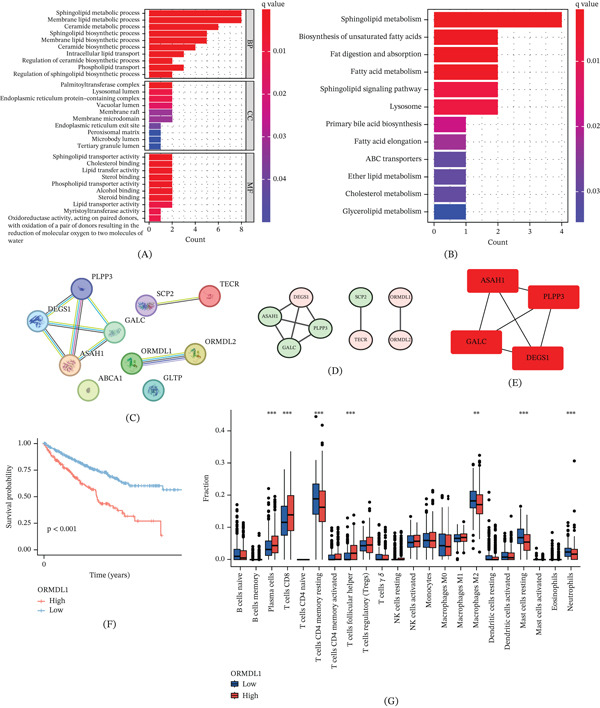
Enrichment analysis and protein interaction network of SRCGs. (A) GO enrichment analysis. (B) KEGG pathway analysis. (C) PPI network of SRCGs. (D) Expression levels of genes in the PPI network. (E) Identification of four core genes within the protein interaction network. (F) Survival analysis of ORMDL1. (G) Immune cell infiltration analysis of ORMDL1.

To explore the relationship between signaling pathways, BPs, and SRCGs, GO functional and KEGG enrichment analyses were conducted on the SRCGs incorporated in the model. Significantly enriched items (*q* − value < 0.05) were selected for further presentation. In the GO enrichment analysis, the most prominent BPs included the sphingolipid metabolic process and membrane lipid metabolic process, while CCs were predominantly associated with the palmitoyltransferase complex and lysosomal lumen. The MFs were primarily characterized by sphingolipid transporter activity and cholesterol binding (Figure 13A). Additionally, KEGG enrichment analysis highlighted the crucial roles of pathways such as sphingolipid metabolism, fatty acid metabolism, and sphingolipid signaling (Figure 13B). In addition, a STRING‐based PPI network was constructed using the modeled SRCGs, showing that most encoded proteins, except ABCA1, GLTP, APOE, and SUMF2, were connected within the interaction network (Figure 13C). Among these, DEGS1, TECR, ORMDL1, and ORMDL2 were identified as genes with upregulated expression, while ASAH1, PLPP3, GALC, and SCP2 were downregulated (Figure 13D). Analysis of protein interaction networks led to the identification of four core genes: ASAH1, DEGS1, PLPP3, and GALC (Figure 13E).

We also conducted survival and immune analyses focusing on the ORMDL1 gene. The survival analysis revealed significant prognostic differences between the high‐ and low‐risk groups, with the low‐expression group demonstrating a better prognosis, confirming ORMDL1 as a risk gene (Figure 13F). The correlation analysis of immune cells revealed a significantly higher abundance of immune cells, such as plasma cells and CD8+ T cells, in the high‐risk group compared to the low‐risk group (Figure 13G).

### 3.13. Mutational Landscape and Burden in TCGA‐KIRC

**Figure 14:**
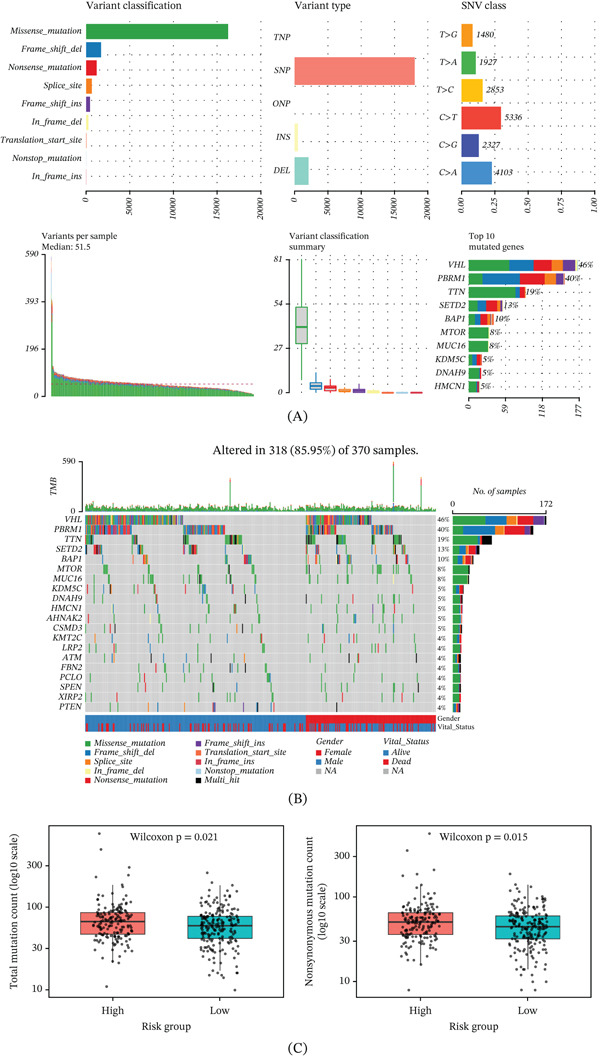
Somatic mutation patterns in ccRCC and their association with risk stratification. (A) Somatic mutation overview in TCGA‐KIRC. (B) Oncoplot of top mutated genes with clinical annotations. (C) Total and nonsynonymous mutation counts in the high‐ and low‐risk groups.

The mutational landscape of TCGA‐KIRC was first summarized to characterize the global genomic alteration pattern. Missense mutation represented the predominant variant classification, single nucleotide polymorphism was the major variant type, and C > T was the most frequent single nucleotide variant class (Figure 14A). Among the recurrently altered genes, VHL, PBRM1, SETD2, BAP1, and TTN showed relatively high mutation frequencies, reflecting the characteristic mutation profile of KIRC (Figure 14B). We next compared the mutation burden between the high‐ and low‐risk groups. The high‐risk group exhibited significantly increased total mutation counts relative to the low‐risk group (Figure 14C). Similarly, nonsynonymous mutation counts were also significantly higher in the high‐risk group than in the low‐risk group (Figure 14C). These findings indicated that patients with elevated SRCGPS tended to harbor a higher somatic mutation burden.

## 4. Discussion

ccRCC arises from the epithelial cells of renal tubules, standing as a prominent entity among renal malignancies [36–38]. Sphingolipid metabolism, involving complex lipid molecules, is integral to cellular architecture and signaling pathways [39]. Aberrations in this metabolic process are closely linked to oncogenesis, tumor progression, and resistance to chemotherapy in cancer patients [40]. Although sphingolipid metabolism′s role has been extensively studied in various tumors, its specific implications in ccRCC remain insufficiently elucidated [41, 42]. This study represents a novel endeavor to systematically investigate the tumorigenic potential of sphingolipid metabolism–mediated TME patterns in ccRCC. Furthermore, it aims to establish an SRG signature model for prognostic prediction in ccRCC patients, employing 101 machine learning techniques on SRCGs derived from NMF‐annotated cell clusters. This innovative approach provides profound insights into the connection between SRGs and ccRCC, particularly concerning signaling pathways, molecular mechanisms, and immunotherapy, thereby contributing to a deeper understanding of ccRCC pathogenesis and offering more precise strategies for personalized treatment, ultimately enhancing patient prognosis and survival.

TME not only governs tumor initiation and progression but also plays a pivotal role in immune evasion, therapeutic resistance, and metastatic spread [43, 44]. A comprehensive understanding of the intricate composition of the TME and its dynamic interactions with tumor cells is crucial for devising effective therapeutic interventions aimed at improving patient outcomes [45]. This study primarily centers on three immune cell types—NK cells, CD8+ T cells, and macrophages. ccRCC frequently originates from the epithelium of the proximal renal tubule, where epithelial alterations, such as epithelial–mesenchymal transition, are critical in tumorigenesis [46, 47]. Consequently, our investigation commenced with epithelial cells, subsequently extending to other CCs within the TME, to elucidate their roles in the pathogenesis and progression of ccRCC.

Sphingolipid metabolism has been implicated in modulating tumor biology, potentially through its impact on cancer immune dynamics [48]. Immune cells, particularly NK cells, T cells, and macrophages, are pivotal in the TME, playing critical roles in tumor surveillance and containment [49, 50]. Within these immune cells, we identified two representative metagene‐defined clusters, annotated by PSAP and ORMDL1, respectively. KEGG enrichment analysis of NK cells revealed that Cluster 0, characterized by ORMDL1 as a metagene, was significantly enriched in thermogenesis and oxidative phosphorylation pathways. Oxidative phosphorylation, a key intracellular energy‐generating process, is vital for NK‐cell ATP production [51]. This pathway′s activation supports NK cells′ energy‐intensive effector functions, including cytotoxic responses [52]. Thermogenesis, an essential process for systemic energy production, enables NK cells to sustain functional efficiency within the TME [53]. The concurrent enrichment of these pathways suggests that this NK‐cell subset may adopt a metabolically active state that could be relevant to ccRCC progression. Compared to non‐SRG‐regulated clusters, SRG‐regulated clusters exhibited heightened average TF expression activity. Notably, in NK cells, ORMDL1 clusters showed elevated transcriptional activity for the TF IRF1. Interferon regulatory factor (IRF) is integral to the transcriptional regulation of immune responses [54]. While IRF1 acts as a tumor suppressor by regulating inflammation, immunity, cell proliferation, and apoptosis [55, 56], it has also been implicated in promoting tumor growth, particularly by suppressing antitumor immune responses and enhancing drug resistance [57]. Additionally, other TFs that facilitate tumor development, such as YY1, FOSL2, and MAZ, are activated [58–60]. We therefore speculate that the ORMDL1‐defined NK‐cell cluster may be associated with enrichment of oxidative phosphorylation and thermogenesis pathways, together with increased activity of TFs such as IRF1, YY1, and FOSL2, a pattern that may be relevant to ccRCC progression. Thus, it is hypothesized that during ccRCC progression, SRGs modulate the TME by activating specific signaling pathways and promoting TFs that drive tumor development.

A similar expression pattern of the TF IRF1 was observed in the ORMDL1‐associated cluster within T cells. There have been studies proving that IRF1 can inhibit T‐cell immune responses and facilitate tumor immune evasion [57]. Interestingly, the ORMDL1 cluster exhibited reduced scores in both exhausted T cells (CD8_exhau) and cytotoxic T cells (CD8_cytoto). Typically, CD8+ T‐cell exhaustion is recognized as a mechanism of tumor immune escape, impairing the antitumor efficacy of these cells [61], while cytotoxic CD8+ T cells are central to tumor immune surveillance [62]. The reduced exhaustion signals observed in the ORMDL1‐defined T‐cell cluster may indicate a relatively distinct functional state compared with the other CD8+ T‐cell subsets. At the same time, the lower cytotoxicity scores suggest that this cluster may be less directly linked to canonical cytotoxic T‐cell programs and instead may reflect a broader immune regulatory state.

Among immune cells, macrophages demonstrated the most robust interaction with epithelial cells. Within the sphingolipid metabolic framework, the PSAP‐defined macrophage cluster exhibited the highest communication strength with epithelial cells. Metabolic pathway enrichment analysis of these macrophage clusters revealed pronounced activation of PSAP+Mac‐C2 pathways, primarily encompassing energy, lipid, and glucose metabolism, underscoring PSAP′s pivotal role in modulating the metabolic state and functionality of macrophages. PSAP, a protein secreted by tumors, serves as a paracrine inhibitor of both primary and metastatic tumor growth [63]. Prior studies have established the antitumor properties of M1 macrophages [64], with lipid metabolism in these macrophages favoring lipid synthesis and the expression of proinflammatory factors [65]. Moreover, glucose metabolism is crucial for the function of classically activated M1 macrophages [66]. The predominance of M1 macrophages in macrophage typing analyses suggests that PSAP‐associated macrophage clusters may be linked to an M1‐like functional state through these metabolic features, thereby potentially influencing the tumor immune microenvironment. These findings suggest that SRGs may be associated with altered immune homeostasis within the TME through links with macrophage polarization, with potential implications for tumor growth and metastasis.

Within the TME, the intricate interactions and functional specialization among diverse cell types are of paramount importance [67]. In the sphingolipid metabolic context, clusters identified across various cell types demonstrated a significant predominance in afferent signaling, indicating an active response to environmental cues, facilitating adaptation, and regulating cellular functions. Conversely, epithelial cells predominantly exert efferent signals, underscoring their central role in ccRCC by secreting signals that regulate and influence the behavior and functions of other cells within the TME. Notably, signaling molecules such as macrophage MIF and bone bridging protein (SPP1) are highly expressed in epithelial cells in the efferent context and within cell clusters in afferent contexts. Prior research has demonstrated that MIF enables tumor cells to evade immune surveillance by inhibiting immune cell function, thereby facilitating tumor cell survival and proliferation within the TME [68, 69]. SPP1 enhances tumor cell adhesion, migration, and invasion through interactions with the CD44 receptor [70, 71]. Consequently, we hypothesize that in ccRCC, epithelial cells function as signaling hubs that shape the TME′s dynamic balance by influencing immune and stromal cell behavior. In response, immune and stromal cells modulate their mechanisms to maintain the TME′s complexity and diversity. Taken together, these observations raise the possibility that epithelial tumor–associated signaling may contribute to reshaping immune and stromal responses in ways that facilitate tumor persistence under immune surveillance.

In our study, the sphingolipid metabolism enrichment profile revealed a striking divergence between the bulk and single‐cell levels. At the bulk level, the normal group exhibited significantly higher enrichment compared to the tumor group, whereas the reverse was observed for many NMF‐derived signatures at the single‐cell‐guided level. This discrepancy is biologically plausible because bulk RNA‐seq reflects an averaged signal across all cell populations and is therefore strongly influenced by cell composition and signal dilution, whereas metagene‐defined cluster scores better capture the relative enrichment of specific tumor‐associated cellular programs [72]. Thus, although global SRG expression may appear reduced in tumors at the bulk level, specific SRG‐related cell subsets can still be preferentially enriched within the TME. These findings highlight how single‐cell‐guided analysis can uncover heterogeneity that is masked in bulk data and provide a more refined view of sphingolipid metabolism–related remodeling in ccRCC. Tumor heterogeneity was further underscored through our comparative analysis with bladder cancer, where cell clusters protective in ccRCC emerged as risky in bladder cancer. This observation not only highlights the unique characteristics of the TME across different cancer types, influencing cell cluster behavior and function, but also suggests that identical cell clusters may activate distinct signaling pathways in different cancer types, resulting in variations in the metabolic environment [73, 74]. This heterogeneity underscores the complexity of the TME and suggests that cell types play divergent roles in the process of different tumors′ development and progression. These findings collectively emphasize the critical role of sphingolipid metabolism and SRG‐mediated TME cell clustering in the progression of ccRCC.

Immune checkpoints serve as crucial regulatory mechanisms within the immune system, designed to avert excessive immune responses and protect normal tissues from autoimmune attacks [75, 76]. Immune checkpoint blockade (ICB) therapy enhances the immune system′s ability to target tumors by activating immune responses, with the enhancement of T‐cell recognition of tumor antigens being a pivotal step. Therefore, the efficacy of ICB therapy is heavily reliant on a robust CD8+ T‐cell response [77–79]. In our study, ORMDL1+CD8+ T‐C6 cells within CD8+ T cells were associated with predicted nonresponse to ICB therapy in ccRCC, a finding further supported by data from a bladder cancer immunotherapy response cohort. The observation that ORMDL1 cell clusters displayed lower scores in both exhausted and cytotoxic CD8+ T cells supports our hypothesis that ORMDL1 primarily regulates other immune cell types within the TME, rather than directly influencing the function of cytotoxic CD8+ T cells. Consequently, ORMDL1 may contribute to the suppression of cytotoxic CD8+ T cells and the development of resistance to ICB therapy in tumors. This intricate regulatory mechanism underscores the necessity for future research to investigate the specific role of genes within particular cell types and their potential implications for immunotherapy.

A prognostic model was developed in this study, incorporating 12 SRCGs: SUMF2, APOE, ORMDL1, ORMDL2, TECR, DEGS1, GLTP, ABCA1, SCP2, ASAH1, GALC, and PLPP3, derived from single‐cell analysis enhanced by machine learning techniques. Evaluation of the calibration curves confirmed the model′s precision and consistency in forecasting ccRCC prognosis, highlighting its potential utility in clinical contexts. Notably, the model exhibited the highest AUC value in the ROC curve, underscoring its superior sensitivity and reliable predictive capabilities, thereby offering clinicians a robust reference and scientific foundation for informed decision‐making in clinical settings.

Compared with prior studies of sphingolipid metabolism in ccRCC, the present work provides a more cell‐state‐resolved framework for prognostic modeling. Earlier studies have shown that SRGs can stratify prognosis in ccRCC and are associated with immune infiltration and drug sensitivity, but these models were largely derived from bulk transcriptomic data and therefore could not fully resolve the contribution of distinct tumor microenvironmental cell states [80]. More recent integrative studies combining bulk and scRNA‐seq have also highlighted the relevance of sphingolipid metabolism in ccRCC, yet the translational value of such signatures still requires further refinement and validation [81]. In addition, several metabolism‐related prognostic studies in ccRCC have demonstrated the predictive relevance of metabolic signatures at the bulk level, but they were also limited in their ability to capture cell‐state‐specific heterogeneity [82, 83]. In contrast, our study derived SRCGs from NMF‐defined metagene‐associated subclusters within immune and stromal compartments, thereby linking sphingolipid metabolism to intracell‐type heterogeneity rather than relying solely on bulk‐level gene selection. Furthermore, by systematically screening 101 machine learning combinations and validating the final model across multiple independent cohorts, our work extends metabolism‐related prognostic modeling beyond conventional bulk‐based signatures and provides a more interpretable framework connecting sphingolipid‐related cellular programs with prognosis, immunotherapy response, and candidate therapeutic vulnerabilities in ccRCC.

The mutation analysis provided additional insight into the genomic background of TCGA‐KIRC. The overall mutational spectrum, together with frequent alterations in canonical ccRCC‐associated genes such as VHL, PBRM1, SETD2, and BAP1, suggested that our cohort retained the representative somatic mutation features of KIRC. Within this genomic context, the high‐risk group exhibited significantly higher total and nonsynonymous mutation counts than the low‐risk group, indicating a heavier somatic mutation burden in patients with elevated SRCGPS. Together, these findings suggest that the high‐risk subgroup was not only embedded in a typical KIRC mutational context but also displayed a significantly increased somatic mutation burden.

The drug sensitivity analysis further extends the potential clinical relevance of the SRCGPS model. By identifying differential predicted IC_50_ profiles between the high‐ and low‐risk groups, our findings suggest that SRCGPS‐based stratification may help prioritize candidate therapeutic agents for distinct patient subsets. Notably, several agents predicted to be more effective in the high‐risk group are directionally consistent with therapeutic strategies already relevant to ccRCC. For example, axitinib remains clinically relevant in advanced renal cell carcinoma [8], while predicted sensitivity to rapamycin may point to a potential role for mTOR pathway inhibition in selected patients [84, 85]. The sensitivity pattern for sorafenib is likewise broadly compatible with antiangiogenic treatment strategies, although its role in current practice is more limited [86]. By contrast, agents such as bortezomib, cisplatin, and dabrafenib are not part of the current standard systemic treatment framework for ccRCC and should therefore be regarded as exploratory candidates [8].

This study provided a detailed characterization of the TME in ccRCC, focusing on distinct cell clusters through a single‐cell NMF approach. This approach enabled a refined characterization of gene expression patterns across various cell types, particularly those involved in immune responses and metabolic processes. Prognostic models were developed using identified metagenes in combination with 101 machine learning techniques, offering novel tools for personalized treatment and prognosis prediction in ccRCC. Our findings provide insight into the potential mechanisms by which these metagene‐defined cellular programs may be associated with cellular functions and interactions within the TME, highlighting the possible role of metabolic regulation in tumor progression. Despite these advancements, the study has several limitations. The reliance on publicly available data may not fully capture patient heterogeneity, and the limited sample size may overlook low‐abundance cells and rare subpopulations. Additionally, as a retrospective analysis, the conclusions are based on existing data, necessitating further validation of clinical relevance and application potential through clinical studies. Future research should focus on deepening the understanding of cellular communication and signaling within the TME, with greater integration of clinical data to improve prognostic stratification and therapeutic decision‐making in ccRCC.

## 5. Conclusion

This study provides a comprehensive investigation of ccRCC by applying single‐cell NMF to characterize the sphingolipid metabolism–regulated TME. By highlighting the critical role of immune cells, it reveals how tumor–immune interactions may contribute to immune evasion in the context of sphingolipid metabolism, thereby offering new perspectives for immunotherapeutic research. In addition, we developed a prognostic model based on 101 machine learning combinations to improve patient risk stratification and outcome prediction in ccRCC. Collectively, these findings deepen our understanding of immune‐related mechanisms and metabolism‐associated therapeutic vulnerabilities in ccRCC, and they may also provide candidate biomarkers and potential therapeutic targets for future biomarker‐guided treatment strategies and functional validation.

## Author Contributions

Conceptualization, Jinbang Huang, Yaojun Zhou, Shi Huang, and Gang Deng. Data curation, Shunsheng Wang and Yaojun Zhou. Formal analysis, Shunsheng Wang, Yaojun Zhou, and Hui Zhang. Supervision, Yanyuan Zhang, Haiwei Su, and Gang Deng. Validation, Hongling Zhu and Yuhan Lin. Visualization, Hongling Zhu and Shi Huang. Writing—original draft, Jinbang Huang, Shunsheng Wang, and Haiwei Su. Writing—review and editing, Jinbang Huang, Yanyuan Zhang, and Gang Deng. Jinbang Huang, Shunsheng Wang, and Yaojun Zhou have contributed equally to this work.

## Funding

This study was supported by Shenzhen Fundamental Research Program (Grant No. JCYJ20220530145006013), the Seventh Affiliated Hospital of Sun Yat‐sen University (the Hospital Key Medical Discipline Construction Program Matching Funds), and a grant from the Sichuan Provincial National Innovation Training Programme for College Students (Grant No. 202410632005).

## Disclosure

All authors have reviewed the final version of the manuscript and have approved to submit it to the journal. All authors have agreed to publish this manuscript.

## Ethics Statement

The authors have nothing to report.

## Consent

The authors have nothing to report.

## Conflicts of Interest

The authors declare no conflicts of interest.

## Data Availability

Publicly available datasets were analyzed in this study, and the download links for these data are described in the Material and Methods section. Moreover, the raw data files for the study can be obtained through the following link: https://www.jianguoyun.com/p/DbpeE6MQlJ2jDRjgm-0FIAA and are available for download.
